# Supplementary figures and images for: Characterization of low-density granulocytes in COVID-19
Source: PLoS Pathog. 2021 Jul 6;17(7):e1009721. doi: 10.1371/journal.ppat.1009721 (PMC8284631; doi:10.1371/journal.ppat.1009721)

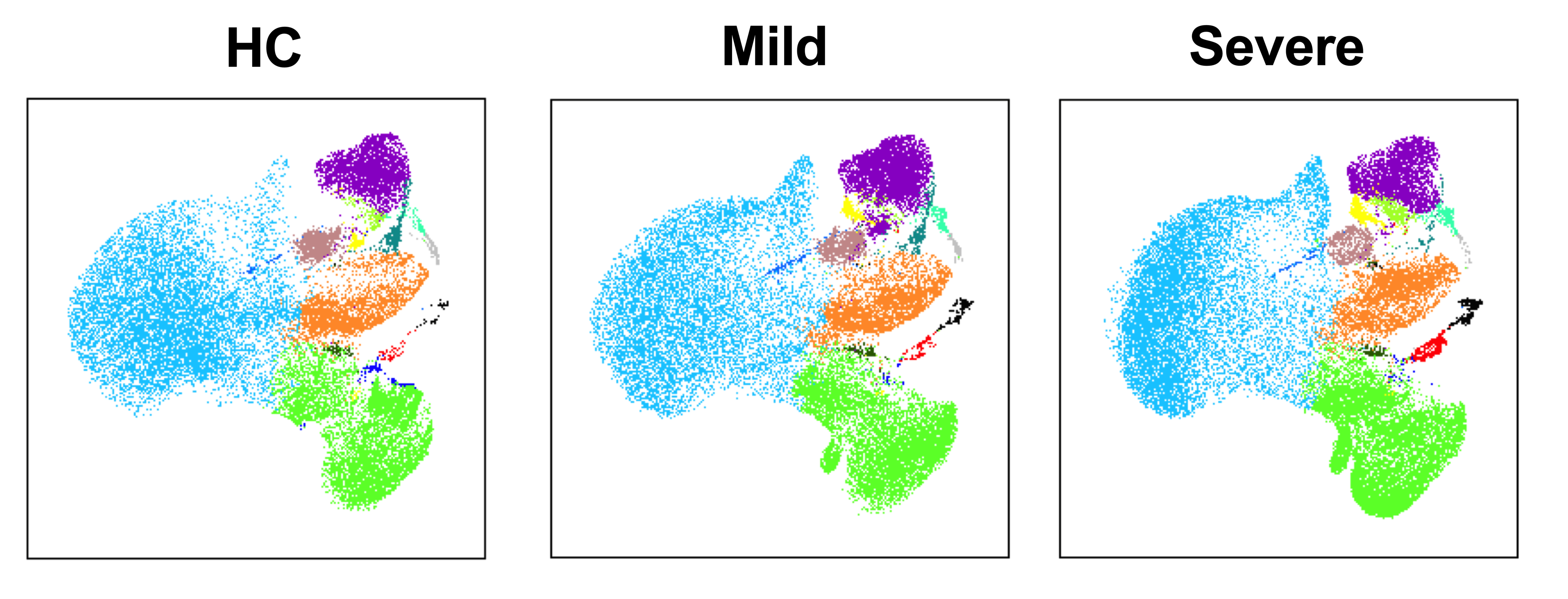

Supplement: S1 Fig — Fresh PBMCs obtained from acute mild (outpatients) and severe (hospitalized) COVID-19 patients as well as HC were stained with a panel of antibodies (see S2 Table for details) and analyzed by flow cytometry. The whole PBMC fractions were dimensionally reduced by UMAP and clustered by FlowSOM. (TIF) [file ppat.1009721.s003.tif]

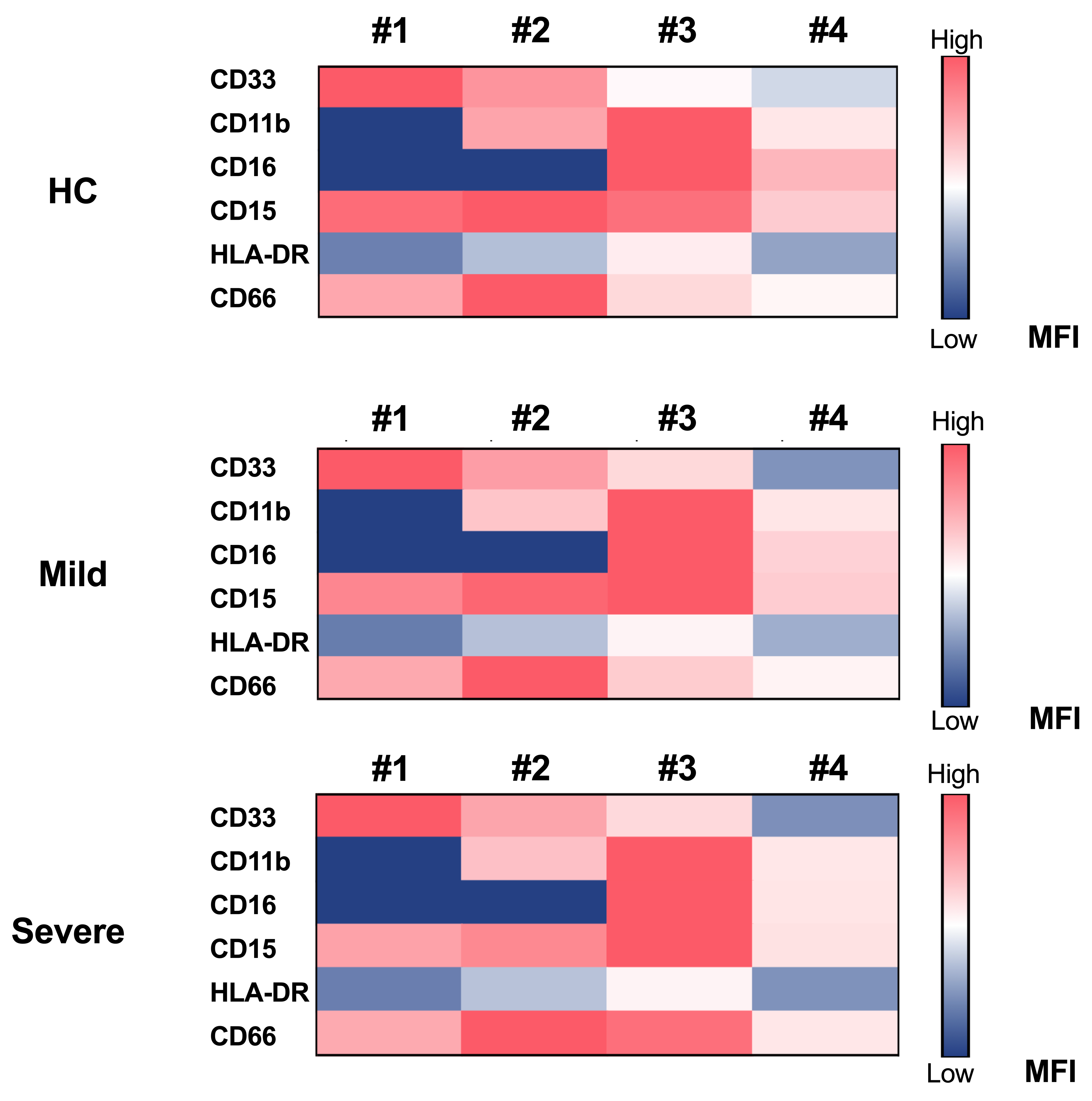

Supplement: S2 Fig — The CD66+CD15+ LDGs formed four different clusters with variable density as identified by UMAP and FlowSOM analysis in HC, mild and severe COVID-19. A detailed heatmap indicating mean MFI of CD33, CD11b, CD16, CD15, HLA-DR and CD66 in each LDG subset (clusters #1-#4) of HC, mild and severe COVID-19. (TIF) [file ppat.1009721.s004.tif]

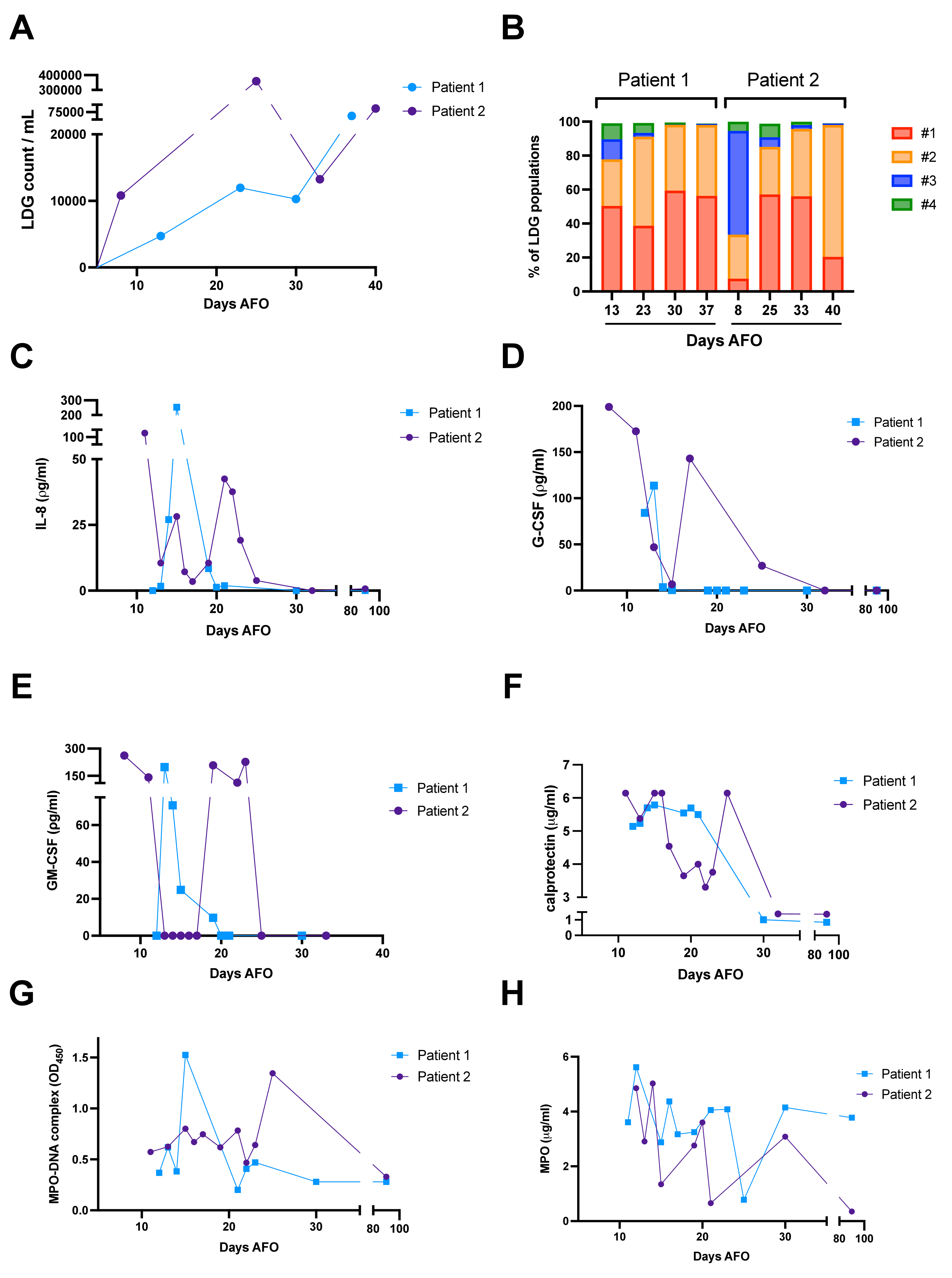

Supplement: S3 Fig — Kinetic analysis of various circulating factors during prolonged hospitalization of two severe COVID-19 patients (patient #1 and #2). (A) LDG counts (calculated similarly as in Fig 1) (B) LDG subset frequencies (same clusters #1-#4 as in Fig 2) (C) IL-8. (D) IL-6. (E) G-CSF. (F) GM-CSF. (G) MPO-DNA. (H) calprotectin. AFO = after fever onset. (TIF) [file ppat.1009721.s005.tif]

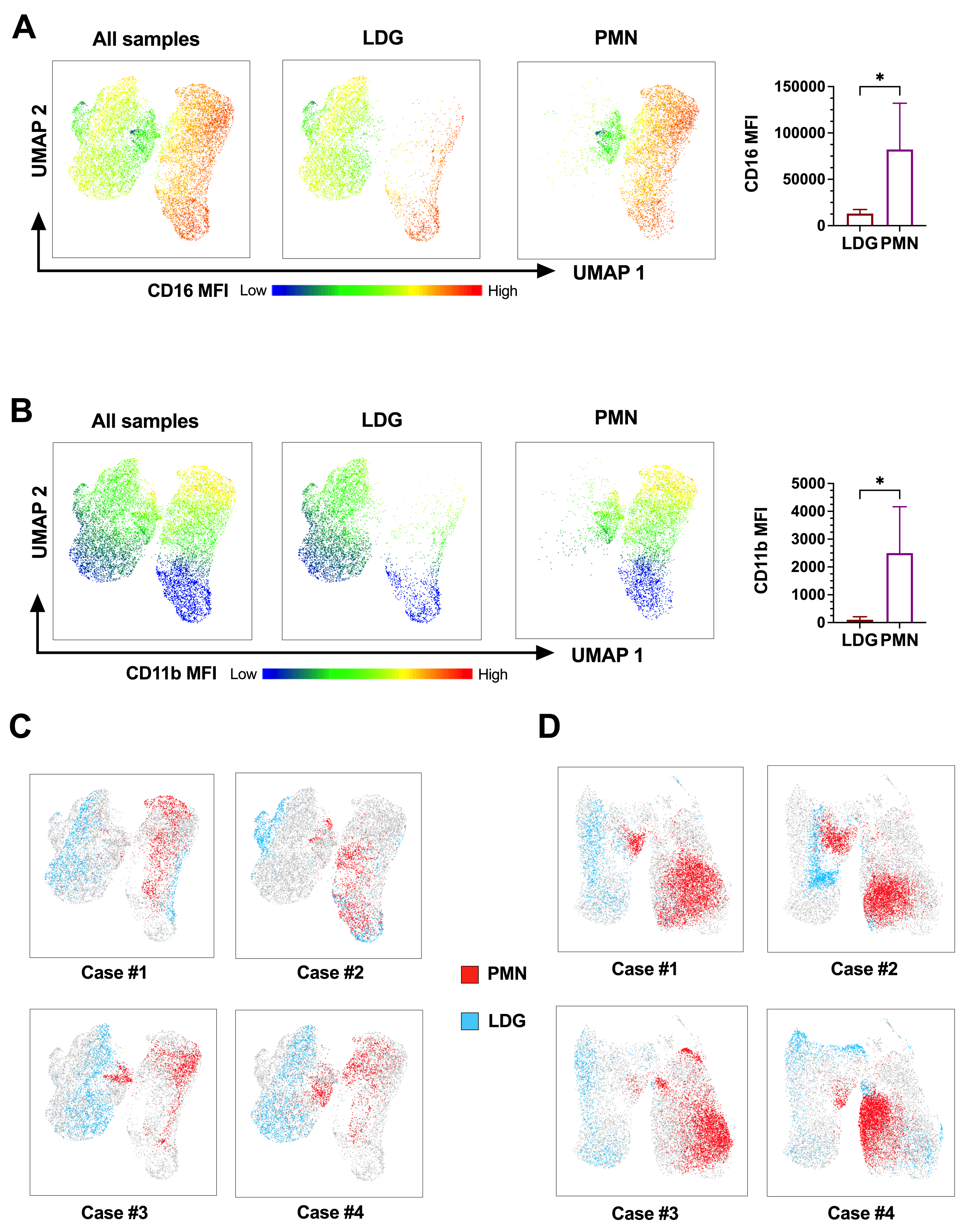

Supplement: S4 Fig — Overlay of CD16 (A) and CD11b (C) MFI heatmaps in dimensionally reduced LDG and PMN population density plots (left) and bar graphs of MFIs of individual severe COVID-19 patients (right). Dimensional reduction was performed by UMAP using CD15, CD66, CD11b, CD16 and HLA-DR MFIs of live CD3-/CD14-/CD19-/CD56- /CD66+/CD15+ population. (C-D) Detailed UMAP localization of CD66+/CD15+ cells from four individual severe (C) and mild (D) COVID-19 patients, isolated from PBMC (LDGs) or PMN fractions. (TIF) [file ppat.1009721.s006.tif]
