# Supplementary material for: Characterization of low-density granulocytes in COVID-19
Source: PLoS Pathog. 2021 Jul 6;17(7):e1009721. doi: 10.1371/journal.ppat.1009721 (PMC8284631; doi:10.1371/journal.ppat.1009721)
Supplement: Attachment — Submitted filename: PLOS-answerstoreviewers.pdf [file ppat.1009721.s007.pdf]

Dear Mr. Strandin,

Thank you very much for submitting your manuscript "Low-density granulocytes are a hallmark of COVID-19 pathogenesis" (PPATHOGENS-D-20-02055) for consideration at PLOS Pathogens. As with all papers peer reviewed by the journal, your manuscript was reviewed by members of the editorial board and by several independent peer reviewers. Based on the reports, we regret to inform you that we have decided to reject the paper due to methodological issues raised by both reviewers. That said, if you were interested in addressing these concerns (which would require an expanded number of patients and controls), we would consider reviewing a "new manuscript." We also would understand if you decide not to do this and submit elsewhere.

Both the Editors and reviewers felt the premise of the paper was interesting and important. However, the reviewers raised significant issues with the current data set and analysis. Reviewer #1 (who was more supportive) raised issue with the low sample size that precluded analysis in Fig 1 of more functional subset linkage to the different stages of disease severity and/or COVID-19 clinical characteristics. He/she also had concerns in Fig 2 about differences (or lack thereof) between non-ICU and ICU patients, and requested additional analysis of NETosis as well as markers of neutrophils activation and function. Reviewer #2 (who was much more negative) had concerns about the classification of LDGs (which I surmise may have some lack of consensus in the field). More importantly, he/she raised concerns about the controls and felt that patients with other infectious diseases are needed for comparison, given the well established left-shift in neutrophil populations that occurs with infections. In other words, how much of the findings are really COVID-19 specific and do you really establish that LDGs contribute to COVID-19 pathogenesis?

The reviews are attached below this email, and we hope you will find them helpful if you decide to revise the manuscript or submit elsewhere. We are sorry that we cannot be more positive on this occasion. We very much appreciate your wish to present your work in one of PLOS's Open Access publications.

Thank you for your support, and we hope that you will consider PLOS Pathogens for other submissions in the future.

Sincerely,

Michael Diamond
Section Editor
PLOS Pathogens

Kasturi Haldar
Editor-in-Chief
PLOS Pathogens
orcid.org/0000-0001-5065-158X

Michael Malim
Editor-in-Chief
PLOS Pathogens
orcid.org/0000-0002-7699-2064

****

Reviewer's Responses to Questions

Part I – Summary: Please use this section to discuss strengths/weaknesses of study, novelty/significance, general execution and scholarship.

Reviewer #1: The manuscript by Cabrera and colleagues identifies a population of low-density granulocytes (LDGs) that are increased in the circulation of patients with COVID-19. They go on to try and phenotype and functionally characterize four different LDG subsets. Strengths include the identification of LDG subsets in non-ICU and ICU patients with COVID-19. Weaknesses include the lack of more functional subset phenotyping and association with different stages of disease severity and/or COVID-19 clinical characteristics. Thus, the findings are more descriptive and observational rather than providing functional insights into the LDG and PMN subsets.

Reviewer #2: The role of neutrophils and neutrophil phenotypes in COVID-19 is timely and important. The authors nicely show that the neutrophil compartment in COVID19 is different when compared with age and sex matched controls.

Part II – Major Issues: Key Experiments Required for Acceptance. Please use this section to detail the key new experiments or modifications of existing experiments that should be absolutely required to validate study conclusions. Generally, there should be no more than 3 such required experiments or major modifications for a "Major Revision" recommendation. If more than 3 experiments are necessary to validate the study conclusions, then you are encouraged to recommend "Reject".

Reviewer #1: 1. Data in Fig. 1B show an interesting spread in numbers suggesting 3 distinct subsets of COVID-19 ICU patients. The subsets may define certain aspects of the disease. Given the low sample size, it is difficult for the authors to say anything about this. Thus, it would be important to increase sample size, if possible, in order to understand what is clinically different between very high % LDGs versus the intermediate and low LDG patients. Other than the ICU status, is the % LDG reflective of different stages or clinical aspects of COVID-19?

Answer: We do agree that our previous submission suffered from relatively low sample size, since we decided to prioritize promptness and try to come out as quickly as possible with our admittedly somewhat preliminary findings. However, in the current resubmission we have addressed this issue by enrolling additional age and sex-matched healthy controls (n=14) and hospitalized COVID-19 patients (n=34). Importantly, we have also included non-hospitalized mild COVID-19 cases (n=21) as a new patient group, which enabled us to make more robust comparisons between patients showing clearly differing disease severity profiles.

We decided also to remove the previously used patient disease severity grouping defined by the need of ICU treatment and replaced this by assessing several different factors reflecting disease severity (level of oxygen supplementation, length of hospital stay, IL-6, calprotectin, etc). The association between LDG counts and individual severity parameters was then analyzed by spearman correlation coefficient and presented in a matrix (Fig. 3G).

2. Although statistical significance was achieved in Fig. 2C between HC and ICU, there appears to be no difference between non-ICU and ICU patients. These findings should be validated and extended with additional NET assays. There are other markers of NETosis.

Answer: We repeated the MPO-DNA complex measurement now with additional patient samples and by different patient grouping (healthy controls vs. mild COVID-19 vs severe COVID-19) to allow for more robust comparisons and improved statistical significance between groups. In this case, MPO-DNA levels were specifically increased significantly in the hospitalized group (Fig. 3E). We have also included additional markers of granulocyte recruitment and activation (IL-8, G-CSF, MPO, calprotectin). We assessed the association of each marker to disease outcome specifically in hospitalized patients by measuring their correlations to various factors reflecting disease severity.

3. Additional markers of neutrophil activation and function should be used in the flow cytometry analysis.

Answer: We added markers in the flow cytometry experiment using newly recruited patient samples to better describe the LDG phenotypes. We included CD33 as a well-defined marker of immature granulocytes and LOX-1 as marker of G-MDSCs.

Reviewer #2: The are several 'main limitations' to this study:

1. The suggestion that LDGs belong to a separate population of neutrophils is not based on hard evidence. The work of Kaplan (who first described this concept in detail) has never put forward good scientific evidence for LDG's as seperate phenotype. The publication by Hassani et al (20) in fact suggests that the shift in density is mainly caused by activation of all phenotypes and that the density 1.077 g/ml (density of Ficoll) is completely arbitrary.

Answer: We fully agree with the reviewer that LDGs cannot be defined as a clearly *different* population from “normal-density PMNs”, but rather represent distinct granulocyte subsets which are most likely also present in the PMN fraction but harbor lower density for various reasons. The evidence by us and others point out that there are at least two reasons for granulocytes to display the low-density phenotype: the main reason from our point of view is granulocyte immaturity (less developed granulocytes are less dense and mononuclear) and the other, as the reviewer also points out, is granulocyte activation that results in cells to lose their “normal” density. Thus, the increased presence of LDGs during a disease reflects an increased granulocytic activation and, subsequently, a replenishment of the decreased granulocyte pools, by an increased early release of immature granulocytes from the bone marrow, as demonstrated by Van Grinsven et al (2019)[1]. Not directly linked to this comment, but we have now analyzed the data also through unsupervised clustering as a more unbiased way of identifying the different LDG subsets.

[1] Van Grinsven, E., Textor, J., Hustin, L. S., Wolf, K., Koenderman, L., & Vrisekoop, N. (2019). Immature neutrophils released in acute inflammation exhibit efficient migration despite incomplete segmentation of the nucleus. *The Journal of Immunology*, 202(1), 207-217.

2. The study is basically a case control study where normal matched volunteers are the control. This can lead to misinterpretation of COVID19 specific findings/mechanisms. This is important as most of the findings in the article fit with the well-known left shift generally seen in many acute diseases such as caused by infections by many micro-organisms. So a case-control study should have been performed with acute diseases other than COVID-19. It is to be expected that most of the data of the current study are also found in other infectious diseases.

Answer: We fully agree with the reviewer. As explained above, the increased circulating LDG counts most likely reflect increased egress from the bone marrow and granulocyte activation. As such, it is likely that their frequencies are increased also in any other disease with pronounced granulocyte involvement. Therefore, we have rewritten our manuscript in a way which does not emphasize the role of LDGs as a hallmark of COVID-19 specifically, but we rather aimed to characterize these cells better to increase our understanding of LDGs in general.

As said, it is likely that LDGs do appear also in other acute microbial infections involving strong granulocyte activation. However, considering the difficulties of assessing LDGs from fresh blood samples of acutely ill and infected patients, its time- and resource-wise not in our reach to analyze such patient samples as a reference group for our current study, unfortunately.

3. The division between ICU and non-ICU is very artificial as most clinical reasons for ICU admittance are based on clinical confounders other than COVID-19 such as cardiovascular risk factors and coagulopathy.

Answer: This is a good point and we decided to remove the ICU classification from the resubmission. We are instead currently assessing disease severity based on several different factors such as extent of oxygen supplementation and length of hospital stay. While all these parameters have their caveats, assessing several of them at the same time at least gives a better overall picture of disease severity.

4. It is really a missed chance not to include/discuss the major risk factors of critical disease that are not necessarily immune driven. An important part of critical disease is caused by tissue (lung) edema, thrombo-embolische complications and coagulopathy. These are all not necessarily mediated by immune mechanisms, but much more mediated by the bradykinin system.

Answer: Yes, admittedly the detailed molecular mechanisms of COVID-19 pathophysiology are still to large extent unresolved and definitely other factors than those directly immune-related play a role. We have rewritten also the discussion part of this manuscript with this in mind. However, since we are not directly studying coagulation or bradykinin system, we feel going too deeply into these phenomena is out of the scope of this manuscript.

5. The data in the article do not support the last and essential conclusion in the abstract. The data do not show that LDG's are major players in COVID19 pathogenesis.

Answer: This is true. Our results do not impeccably show that LDGs are major players in COVID-19 pathogenesis. We have rewritten the last sentence in the abstract as follows: “Taken together, our data confirms a significant granulocyte activation during COVID-19 and suggest a role for LDGs in COVID-19 pathogenesis.”

Part III – Minor Issues: Editorial and Data Presentation Modifications. Please use this section for editorial suggestions as well as relatively minor modifications of existing data that would enhance clarity.

Reviewer #1: 1. Based on data in Fig. 3A, there are few CD16bright LDGs in the late acute phase of disease. It is thus unclear how the authors can really assess and compare functional changes in this cell population based on the number difference (Fig. 4). In order to say anything specific about this cell population, the authors would need to sort them and then perform functional assays.

Answer: True, the total LDG fraction in the late acute stage (now called convalescent to clearly distinguish it from the strictly acute phase) is less immunosuppressive than the total LDG fraction in the acute stage and as the reviewer suggests, we also think this functional difference might be due to differences in LDG subset composition in the two distinct disease phases. Thus, the data indirectly

suggests the CD16bright (now simply referred to CD16+ for clarity) LDGs are functionally more immunosuppressive than CD16- LDGs.

We have also FACS-sorted different LDG subsets in the current manuscript, but unfortunately, cell numbers retrieved after this process were not enough to assess their functionality. However, this method allowed us to assess LDG developmental stages, demonstrating the nuclear morphology of each of the cellular subsets, matching their marker phenotype.

2. Data in Fig. 3B is from two mechanically ventilated patients. Are these findings specific to mechanical ventilation?

Answer: Without an appropriate non-mechanically ventilated control group at the same phase of disease we cannot really assess how LDG responses are mediated by mechanical ventilation. We admit this and in the current manuscript our intention is not to claim that the samples obtained from patients with prolonged mechanical ventilation would be representative of COVID-19 in general (this data is now presented as a supplement). However, since the LDG subset composition in these patients differs significantly from those obtained at early acute stage, we feel we can use these samples as a reference group to study the functionality of LDGs.

3. There are numerous conclusions made in the Discussion with very limited experimental support. These should be toned down.

Answer: The discussion has been fully rewritten, as well as the results. Figures have also been improved significantly.

4. NETosis was not really studied, only the formation of MPO-DNA complex by ELISA. Findings need to be expanded here in order to understand potential differences between NETosis by PMN versus LDG in the context of COVID-19.

Answer: We have added several additional markers of granulocyte migration and activation (IL-8, G-CSF, MPO, calprotectin) in the current manuscript to get a better overall picture of granulocyte activation in our patients. Since assaying NETosis directly from patient blood samples is challenging, we are now putting less emphasis on NETosis but instead discuss granulocyte involvement and activation from a broader perspective (including NETosis, degranulation, recruitment, migration) in the current manuscript.

Reviewer #2: (No Response)
