# Supplementary material for: Characterization of low-density granulocytes in COVID-19
Source: PLoS Pathog. 2021 Jul 6;17(7):e1009721. doi: 10.1371/journal.ppat.1009721 (PMC8284631; doi:10.1371/journal.ppat.1009721)
Supplement: Attachment — Submitted filename: Letter-editor.pdf [file ppat.1009721.s008.pdf]

Dear Mr. Diamond,

We appreciate the review by the members of the editorial board and the independent reviewers, and your consideration to rule in favor of publication. For this reason, we have responded the issues raised by yourself and the reviewers. We adapted the manuscript in a way that your requests would be fulfilled. The changes made to the manuscript are listed as independent responses to each of your petitions, and further details can also be found as responses to the reviewer's concerns.

1. You agree on the fact that LDG may not comprise of a separate phenotype of neutrophils, yet you persist with implying that LDG are such a population in the Abstract and Discussion. You also neglect to cite studies that do not support the main hypothesis that neutrophils are important in the initiation of disease up to the moment patients have to go to the ICU. These points need to be addressed.

    - LDGs are no longer called a distinct subset of circulating neutrophils in this manuscript. We deemed useful to refer to them as *granulocytes of lower density* on some occasions, to avoid the misconception of LDGs being a different type of neutrophils. Their isolation from PBMC has been highlighted more than in the previous version, and their presence in this fraction continues to be explained by their mere lower density, which can arise from an immature mononuclear or hyposegmented nucleus and/or by a loss of density of some mature PMNs, which we hypothesize could arise from activation.

2. Discussion about cause and consequence. Neutrophils might still be guilty by association. This is not carefully addressed in the Discussion.

    - We have rewritten the parts of the discussion that were misleading by suggesting a role of LDGs in the initiation of the disease (or pathogenesis). This idea has been redirected towards neutrophils being actors during the general pathophysiology of COVID-19, contributing to *disease progression*. This goes in line with their proposed role as modulators and suppressors of the adaptive immune response, given their demonstrated suppressive effect in T lymphocytes.

3. A left shift during inflammatory conditions associated with the occurrence of progenitors in the peripheral blood is expected. This is normal physiology and not necessarily pathophysiology and accordingly should be acknowledged.

  - The 'guilty by association' issue has been addressed by detailing the emergency myelopoiesis as a response of inflammation, which is why we support the idea that LDGs in infectious processes are indeed a product of a 'left-shift', instead of being exclusive for COVID-19. Spijkerman *et al.* (2021) has been added as a reference in this matter, and their findings on a lack of activation of neutrophils at hospital presentation was discussed.

4. The complex multidimensional data supporting the presence of progenitors in the peripheral blood is not much better than the 2D plots on the expression of CD11b and CD16. You do not acknowledge Hidalgo's paper in Trends in Immunology (ref 26) that already used this gating strategy. This acknowledgement should be clearly made.

  - Hidalgo *et al.* (2019) and Pedersen *et al.* (2016) have been properly acknowledged for the CD11b/CD16 sorting strategy to assess the different maturation stages.

Responses to reviewer's comments:

- Reviewer: although the authors "fully agree" on the issue that LDG'S are not a specific subset but either young and/or activated neutrophils, they persist throughout the article with the suggestion that LDG comprise of separate subsets of neutrophils (1-4) while the data merely show a left-shift. The choice of sorting on CD11b/CD16 and finding (pro)myelocytes, meta's and mature cells is not new and shown before in ref. 26 Hidalgo et al. This should be acknowledged.

  - *Our aim was to characterize the phenotype and function of low-density granulocytes (LDGs) in acute COVID-19. LDGs are defined as granulocytes isolated from the PBMC fraction, rather than whole blood directly. Thus, we are aware that we are not describing all neutrophils during COVID19 and find the need to specify it. We centered this study around those granulocytes of lower density that separate from normal-density neutrophils during Ficoll gradient. It is a fact that LDGs have been for a while considered a distinct subset of granulocytes in other diseases, and the bibliography is diverse depending on the disease in question. However, in this study we intend to shed light into these*

so-called "LDGs" during infectious processes, since they have not been well characterized in this field. We agree that in these circumstances of an acute inflammation they are increased as a result of emergency myelopoiesis, with an premature release of immature granulocytes into the circulation. We confirm this by assessing the stages of development with maturation markers, a sorting strategy that has indeed been used before by Hidalgo et al (2019) and Pedersen et al (2016). Both studies have now been acknowledged in the discussion.

- Reviewer: the issue of the difficulties obtaining relevant disease controls is well taken, but should be better discussed in terms of left shifts in other acute infectious diseases.

    - *We understand and have broaden the discussion concerning emergency myelopoiesis as a physiological response to inflammation and/or infection. This left shift has been acknowledged and the study by Spijkerman et al. (2021) comparing COVID-19 neutrophil compartment response to other bacterial and viral infections has been made. We believe this goes in line with demonstrating that during acute inflammatory processes such as infections the increase in neutrophils of lower density comes from the premature release of granulocyte precursors, which should also be taken into consideration during chronic pathological processes where LDGs are better defined and are still considered a different subset of cells compared to their normal- density counterparts.*

- Reviewer: the issue here was that the alternative pathogenesis of COVID19 might drive the disease including causing tissue damage through thrombo-embolisms and reperfusion injury. More severe disease would then lead to more release of DAMP's leading to more activation of the neutrophil compartment. So the left shift can be the consequence of disease rather than the cause. In fact, Spijkerman et al. (JLB 2021) supports this hypothesis by not finding much indications of an activated neutrophil compartment in COVID19 patients during hospital admission. This study is ignored but at least the concept should be discussed.

    - *This is a good point. The idea that neutrophils play a role during COVID-19 pathogenesis is basically supported by the fact that these cells are part of the innate immune response, which is the first responder against PAMPs. However, more recent studies such as this of Spijkerman et al. (2021) do suggest the granulocyte expansion and activation (described in numerous COVID-19 articles) to be a response to other pathophysiological events that take place in this infection. For this reason, we have rewritten the parts of the text that suggested the role played by LDGs to be relevant during the initiation of disease and we cited the aforementioned paper. Additionally, we avoided using the word 'pathogenesis', which could be misleading. Instead, we redirected the focus towards disease progression and neutrophils being actors during the general pathophysiology of COVID-19, in which they are proposed as*

modulators and suppressors of the adaptive immune response, given their demonstrated effect in T lymphocytes.

- Reviewer: See last issue: the data in the literature not all support the concept of activation of granulocytes in COVID19 at hospital admission (initiation of disease). It is important to establish whether activation of neutrophils is associated with the cause or consequence of disease. The issue of guilty by association rather than causation is still not adequately addressed.

  - *As explained in issue 4, neutrophils are no longer addressed as part of the pathogenesis. Whether they are cause or consequence is still to be established and more studies are needed. Thus, we described LDGs as actors during the pathophysiology of COVID-19, possibly contributing to the progression of the disease. Our functional assays suggest this role to be that of MDSCs.*

- Reviewer #2: The figures are low-quality in this submission.

  - *The images have now been exported from TIFF to TIFF instead of PDF to TIFF, to ensure high resolution when crossing platforms.*

We hope these modifications are in line with the requests made, as well as the answers to the reviewer's questions. If further changes are needed, do not hesitate to inform us and we will make sure to comply with the demands.

Kind regards,
Luz Cabrera & Tomas Strandin
